# Electrical impedance tomography: Amplitudes of cardiac related impedance changes in the lung are highly position dependent

**DOI:** 10.1371/journal.pone.0188313

**Published:** 2017-11-16

**Authors:** Michael Graf, Thomas Riedel

**Affiliations:** 1 Division of Paediatric Pulmonology, Department of Paediatrics, Inselspital, University Children’s Hospital and University of Bern, Bern, Switzerland; 2 Laboratory of Nanoscale Biology, Institute of Bioengineering, School of Engineering, Ecole polytechnique federal de Lausanne, Lausanne, Switzerland; 3 Department of Paediatrics, Cantonal Hospital Graubuenden, Chur, Switzerland; University of Minnesota, UNITED STATES

## Abstract

**Background:**

Electrical impedance tomography (EIT) is used on the thorax to measure impedance changes due to the presence of air and blood in the lung. This experimental study was performed to investigate the effect of posture on cardiac and respiratory related impedance changes.

**Methods:**

EIT measurements were performed on 14 healthy subjects in left-, right lateral, prone, supine and upright positions. Simultaneously, tidal volume was recorded with an ultrasonic flowmeter. For image reconstruction, the classic Sheffield back-projection and three variants of the modern GREIT algorithm were applied with two different reference frames. Amplitudes of cardiac- and respiratory impedance changes were extracted and compared between the positions.

**Results:**

We found significant differences in both cardiac and respiratory amplitudes between postures. Especially, supine and upright positions showed dramatic changes in amplitude. These differences between postures were unaffected by the change of reference frames in all reconstruction methods except of the classic Sheffield back projection. Possible sources that explain the observed posture dependency are discussed.

**Conclusion:**

Researchers and clinicians need to be aware of this phenomenon when comparing EIT amplitudes in different body positions.

## Introduction

Electrical impedance tomography (EIT) has become increasingly important for bedside monitoring of ventilation distribution [1]. Typically, an array of electrodes along an electrically conductive body injects small currents and measures voltage differences. Mathematical transformations then lead to a tomography image that represents impedance change distributions across a slice of the body. Major changes in resistivity inside the measured tissue, like air-filling of the lungs, can be located and quantified by EIT [2]. During tidal breathing, impedance changes have a linear relationship with changes in lung volume [3]. At the thorax, not only the respiratory induced impedance change can be measured, but also cardiac related changes within the heart region and lung tissue can be observed [4]. These signals, however, are about one order of magnitude smaller than respiratory related impedance changes and were attributed to changes in blood volume in the lungs, i.e. changes of cross-sections of larger vessels due to the pulsatile pressure waveform, movement of tissue or the velocity related conductivity of blood [5]. It is well established that body position influences regional ventilation distributions [6,7]. Ericsson et al demonstrated the effect of belt and body position on impedance changes [8]. However, the combined effect of body position and reconstruction algorithm on the amplitude of the EIT signal itself has not yet been studied.

In a clinical setting EIT measurements are performed on spontaneously breathing or mechanically ventilated subjects. The signals obtained are thus mixtures containing both cardiac and respiratory components. Consequently, it is important to have tools available that allow decomposing this signal into its parts. Currently, four major methods have been proposed to separate cardiac from respiratory signals: Electrocardiography (ECG)-gating, breath-hold, Principal Component Analysis (PCA)-based and frequency-based methods [9].

The most straightforward method relies on filtering of the mixed signal digitally. Typically, this is performed in the frequency domain using Fourier transforms [10]. After separation in the frequency domain the cardiac activity related signal might still suffer from interferences of the impedance changes due to pumping of the heart or due to the fact that the heart rate is locked into the respiratory rate. The movement of the heart and the subsequent deformations of neighboring structures complicate the removal of such kind of interference.

In the present study we apply a modified cross-correlation algorithm to compare the amplitudes of cardiac and respiratory related impedance changes within the lung in different body positions in healthy adults. From observations in a previous study [11], we hypothesize that these amplitudes are significantly different between body positions not explained by physiological changes alone. Additionally, we evaluate the impact of different reconstruction algorithms on these values.

## Materials and methods

### Study design

EIT measurements were performed in 14 healthy non-smoking volunteers (7 females) aged 24 to 54. Subjects were recruited from the hospital staff and were approached by a member of the study team between October and November 2013. Complete measurements could be obtained from all subjects. No additional demographic characteristics were recorded. Subjects were placed in upright, supine, prone, left- and right-lateral positions. All subjects were breathing through a mouthpiece and an ultrasonic flow meter to measure flow and volume during tidal breathing. A nose-clip ensured complete obstruction of the nasal airways. Body positions were applied in random order. Randomization was performed using sealed envelopes. Every measurement consisted of ~45 seconds of spontaneous breathing and ~15 seconds of apnea. Measurements were recorded in triplicates. All results reported are mean values of the three measurements. A recording session lasted for about one hour. During this time subjects were at rest. The local ethic committee (Kantonale Ethikkommission Bern) approved the study. All subjects gave written informed consent.

### Measurements

#### Electrical impedance tomography

A GoeMFII EIT tomograph (CareFusion, The Netherlands) was used with a frame rate of 44 Hz. 16 Ambu^®^ Blue Sensor T self-adhesive electrodes (Synmedic, Switzerland) were equally distributed around the chest at the xyphoid level. An additional reference electrode was placed on the abdomen.

#### Spirometry

In order to synchronize the EIT signal with the flow, we connected an ultrasonic flowmeter (Spiroson Scientific, Ecomedics, Switzerland) to the analog input of the GoeMFII EIT device. This ensured sampling frequency matching of both signals. The voltage differences through the coaxial cable were between -1 and +1V corresponding to the voltage input limits of the GoeMFII (no saturation occurred at normal tidal breathing). The software provided by the manufacturer (Spiroware 2.0) and a 500ml syringe was used to calibrate the flowmeter at the beginning of each measurement day. A low resistance bacterial filter (Hygrovent S, Medisize, The Netherlands) was placed between the subject and the flow head. The values obtained through the analog input were calibrated to correspond to the ones obtained using Spiroware 2.0. Typically, calibration involved offset correction and scaling of the values. The respiratory volume curve was determined by cumulative trapezoidal numerical integration of the flow signal (MATLAB 2013b (MathWorks, Natick, Massachusetts, U.S.A.).

### Data analysis

#### Reconstruction

Reconstruction was dynamic, i.e. impedance differences from a reference were used as a basis for reconstruction. Reconstruction was performed in duplicate using two different reference frames. First, the reference frame was defined as the mean of a stable phase of the first scan in upright position during spontaneous breathing (“Global reference”). Second, the reference frame was defined as the mean of a stable phase of the first scan within each body position during spontaneous breathing (“Local reference”). We used different reconstruction methods, in order to rule out any artifacts introduced by the reconstruction algorithm itself. First, the original, proprietary algorithm of the GoeMFII EIT system was used (R_G_). Here, reconstruction is performed using a weighted back-projection algorithm to map the impedance differences onto a 32x32 pixel matrix of cylindrical shape [12]. Uniform background is assumed, which might provoke misleading results [13]. Additionally, we used a more sophisticated and modern reconstruction technique: the Graz consensus Reconstruction algorithm for EIT (GREIT) [14], included in the Electrical Impedance Tomography and Diffuse Optical Tomography Reconstruction Software (EIDORS) package [15]. In this approach, a detailed finite element model of an adult thorax is used. Reconstruction accuracy can be improved by using prior conductivity information about the probable locations of the inner organs of the chest [16]. Three thorax shaped finite-element models (FEM) were used: One containing a uniform conductivity (R_0_), one considering a lung region of 30% of background conductivity (R_L_), and finally one containing the lung region of 30% as well as a heart region of 200% of background conductivity (R_HL_).

#### Separation of cardiac and respiratory related impedance changes

All data analysis described next was implemented in MATLAB 2013b (MathWorks, Natick, Massachusetts, U.S.A.). After reconstruction, the raw impedance values were converted to a three-dimensional matrix format: 32x32xN, where N is the number of frames of the movie. First, the impedance data was manually separated into spontaneous respiration and apnea phases. Afterwards, the respiration data was roughly filtered to accentuate cardiac and respiratory characteristics respectively. This was achieved using a bandpass filter with predefined cutoffs of 2 and 40bpm for the respiration and 40 and 400bpm for the cardiac activity. Once the two roughly filtered signals were obtained, we searched the peak frequency of respiration and cardiac activity in order to refine our bandpass window. Matlab’s built in *periodogram* function was used to construct the frequency power spectra of the two signals. The maximal peaks of the two power spectra were then defined as the respiratory rate f_RR_ and the heart rate f_HR_. Sometimes, higher order harmonics of the respiratory rate interfere with the heart rate peak. Therefore, all heart rate peaks were visually inspected to ensure correct detection. Those newly obtained peak frequencies are then used to refine the temporal filtering of the impedance data. Briefly, the raw data was bandpass filtered between 0.5*f and 2.5*f, where f corresponds to the peak frequency of either the respiratory rate f_RR_ or the cardiac rate f_HR_.

Three filtered impedance movies were calculated:

A respiratory domain filtered movie using cutoffs of:
  0.5 * f_RR_ > f < 2.5 * f_RR_
A cardiac domain filtered movie using cutoffs of:
  0.5 * f_HR_ > f < 2.5 * f_HR_
A more stringent cardiac domain filtered movie using cutoffs of:
  0.8 * f_HR_ > f < 1.2 * f_HR_

The signal in the heart domain was subject to noise not corresponding to the pulsatile period of the heartbeat. The strictly filtered signal is used to automatically detect start- and endpoints of the pulsatile period. Amplitudes were calculated using the less strictly filtered cardiac signal. In contrast, respiratory domain signals didn’t require strict filtering due to their steady period.

A custom written code was used to extract individual breaths and heart beat signals from the global signal. The mean pixel value within the region of interest (lung) was calculated for every frame. First and second derivatives were then used to find the minima and maxima of the signal. The amplitude of a particular breath or heartbeat was defined as the change in impedance value between its minima and maxima. The following amplitudes were extracted:

**ΔZ**_**CR**_: Cardiac related impedance changes during respiration extracted by filtering the respiration signal in the heart rate domain.
**ΔZ**_**RR**_: Respiratory related impedance changes during respiration extracted by filtering the respiration signal in the respiratory domain.
**ΔZ**_**CA**_: Cardiac related impedance changes during apnea extracted on the untouched signal obtained during apnea.
**V**_**T**_: Tidal breathing extracted from spirometer.

We had one amplitude value per breath or heart pulse for each subject, position and replicate. The values were then first averaged over all breaths/pulses of a given subject, position and replicate. Finally, the reported values were mean values over the triplicate, leaving two amplitude values (cardiac and respiratory) per subject and position. Units of impedance changes were arbitrary, i.e. different reconstruction methods produced different orders of magnitude. To facilitate presentation, we multiplied values obtained through reconstruction R_G_ with 1000. In order to compare respiratory amplitudes between subjects, we normalized respiratory related impedance changes to tidal volumes **ΔZ**_**RR**_ / **V**_**T**_.

#### Spatial separation of heart and lung

In order to spatially separate the impedance change due to the heart from cardiac activity related impedance changes and ventilation we exploited a result described by Grant et al. [9]: the authors found that the impedance changes due to the heart itself are subject to a phase shift (150°-180°) with respect to cardiac related signals inside the lung. This cross-correlation method has also previously been used by different authors for the elimination of the heart region [17,18,19].

In a first step, we defined a group of pixels that corresponded to cardiac related impedance changes inside the lung. To do so, we determined the pixel with the maximum impedance change within the lung domain filtered image. This ensured that we chose the reference inside the lung. The 4-connected pixels from the maximal value made up the reference region.

**Fig 1:**
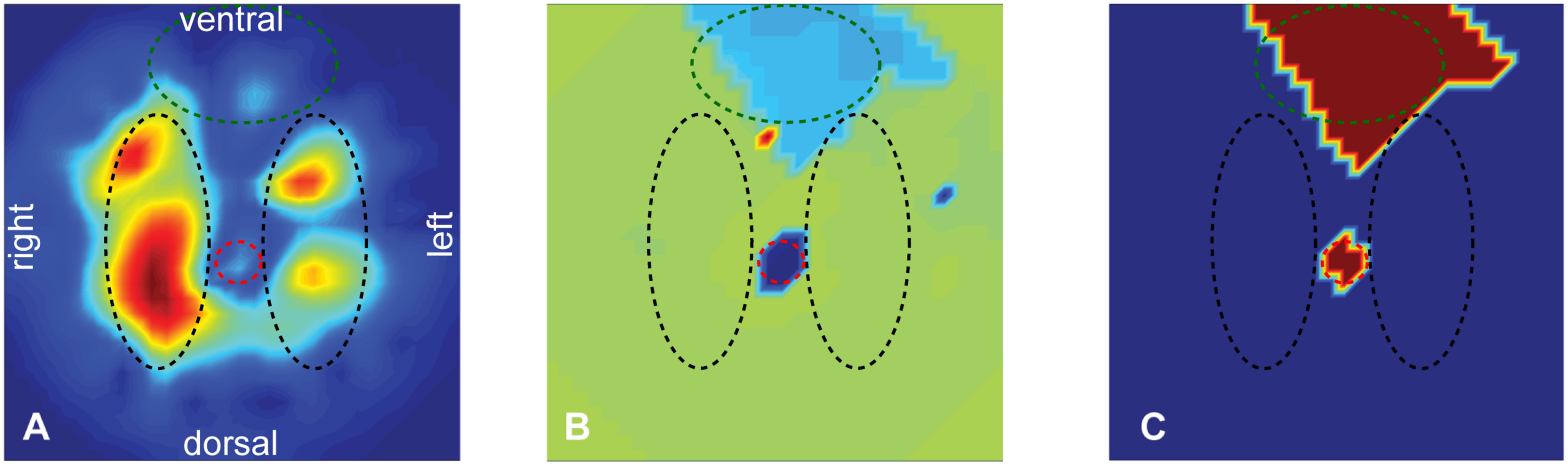
Heart mask. A) The standard deviation over the length of the recording was calculated for each pixel. Regions of high impedance change are in red, regions of low impedance change in blue. The region with the highest impedance change corresponds with a high probability to lung tissue and was used as a reference for cross-correlation. B) Phase image depicting the obtained phase after cross-correlating all pixels with the reference region. Red corresponds to a phase shift of about 150° whereas blue denotes -150°. C) Binary heart mask obtained after thresholding the phase image at ±30° and applying morphological operators. The dashed lines illustrate the different anatomical regions: lung (black), heart (green) and great vessels (red).

In a second step, the mean signal of the reference location was correlated with the time trace of all other pixels. The distance of the maximal correlation to the zero lag point corresponds to the phase shift. The phase shift of each pixel was then converted to a phase angle. In a last step, all pixels that had more than ±30° shift were considered to be due to the heart itself. Using this threshold, a binary image specifying the location of the heart was then created. In order to achieve a coherent heart shape, the binary image was treated with closing and opening operations in order to fill holes and remove isolated single pixels [20]. Fig 1 illustrates the main steps described above.

#### Filter effect on amplitude

To assess the effect of extracting the cardiac related signal from the mixed signal, we compared ΔZ_CR_ and ΔZ_CA_.

#### Filter design

A band-pass finite impulse response (FIR) filter was implemented to filter the individual pixels in lung and heart domain. This filter was used for all filtering purposes described here. In comparison to infinite impulse response (IIR) filters, the FIR has two crucial advantages: It is always stable and it has a linear phase response. The filter is applied in a forward-backward manner in order to improve performance at the edge and remove lag [21]. The bandpass filter was constructed using Matlab’s *fdesign*.*bandpass* function.

#### Statistics

Anderson-Darling test was used to test data for normality. The non-parametric Friedman’s test was employed to compare the different groups of interest. Each block in the test corresponded to a subject, whereas the columns corresponded to the test repetitions, i.e. body positions. A block contained the mean amplitude of the triplicate. In cases where Friedman’s test picked up a significant difference between at least one group with at least one other group, post-hoc analysis after Conover [22] was performed to determine which groups are significantly different. Significance was defined at α = 0.05 after correction for multiple comparisons.

The Wilcoxon signed rank test for paired data [23] was used to assess whether extracting cardiac related impedance changes by filtering the signal during respiration influences the amplitude significantly compared to raw amplitudes of the apnea phase. Pearson correlation coefficient was calculated between changes in heart rate and changes in amplitude between upright and recumbent positions to test for heart rate dependency.

## Results

### Cardiac related impedance changes

**Fig 2:**
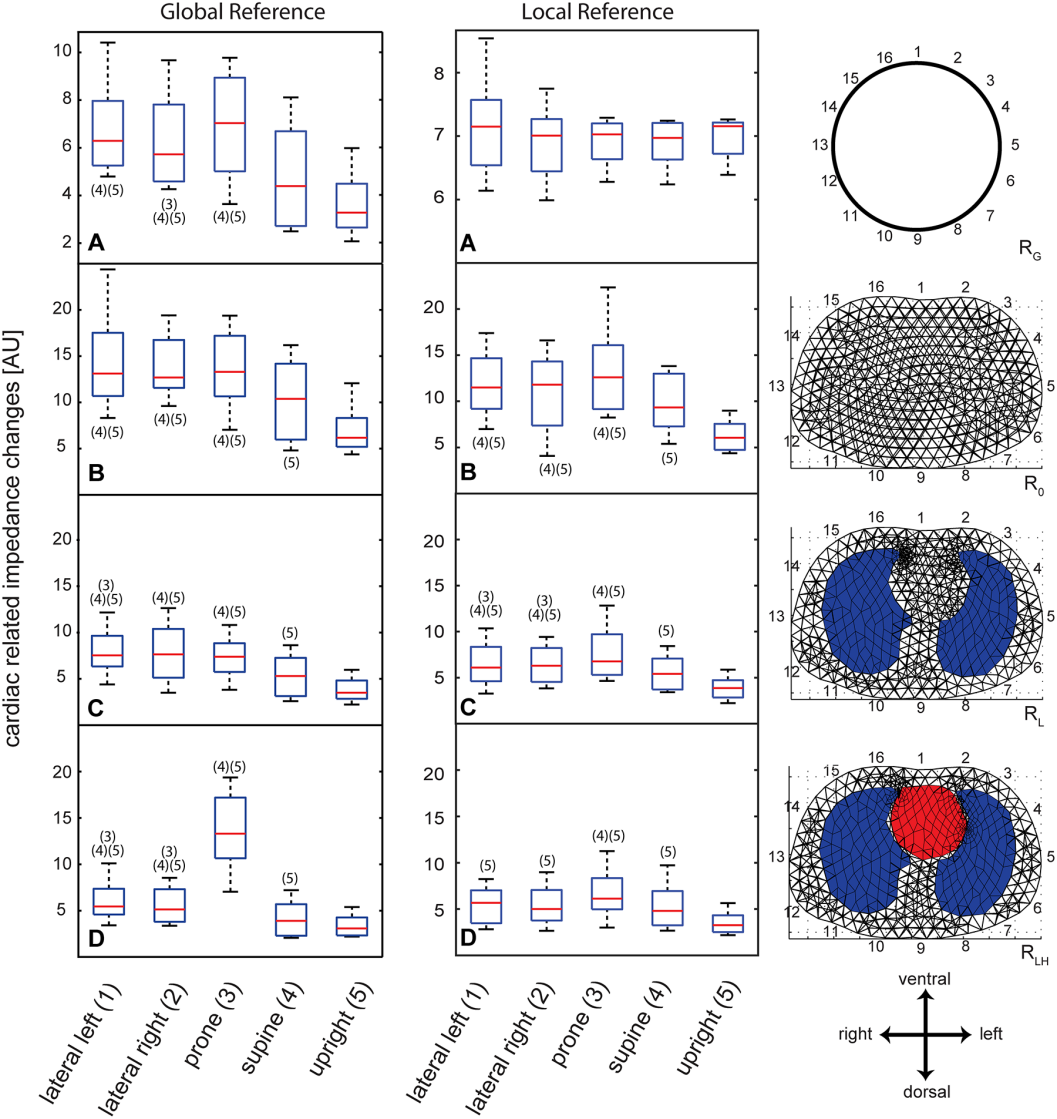
Cardiac related impedance changes. Left side: Boxplots of amplitude levels of cardiac related impedance changes: Image reconstruction referenced to upright position (“Global reference”). Middle: Boxplots of amplitude levels of cardiac related impedance changes: Image reconstruction referenced to the corresponding body position (“Local reference”). Right: Depiction of the corresponding reconstruction algorithm. The cylindrical uniform shape describes the back-projection used with the software provided with the GoeMF II tomograph (A) whereas thorax shaped meshes were used with GREIT reconstruction (B-D). The red lines denote median values, the blue box encompasses values between the 25^th^ and 75^th^ percentile. Whiskers cover 95% of the data. Positions that are significantly different (Friedman’s test) are labeled with their appropriate number.

Cardiac related amplitudes were about an order of magnitude smaller than respiratory related amplitudes. We first calculated the amplitudes of cardiac related impedance changes of the R_G_ reconstruction. The upright and supine positions yielded a significant decrease (p<0.05) in amplitude with respect to left, right lateral or prone when image reconstruction was referenced to upright position (Fig 2 left panel, A). No difference was found between left lateral and prone position. Additionally, significant differences were found between right lateral and prone. When image reconstruction was referenced to the corresponding body position no differences in amplitudes between the different postures could be demonstrated (Fig 2 middle panel, A).

Next, we used different reconstruction algorithms to determine their effect on position-dependence of the cardiac related signals. Independent of the reconstruction method used, the main trend in the data was conserved, i.e. the upright and supine positions showed significantly lower amplitudes than the other postures. These findings were basically unaffected by the reference method used. (Fig 2A–2D). Differences in amplitudes between upright and recumbent positions revealed only very weak or weak negative correlation with differences in heart rate in R_0_, R_L_ and R_LH_ (Pearson’s coefficient -0.16 –-0.28). R_0_ revealed a moderate positive correlation (Pearson’s coefficient 0.46).

### Filter effect on amplitude

**Fig 3:**
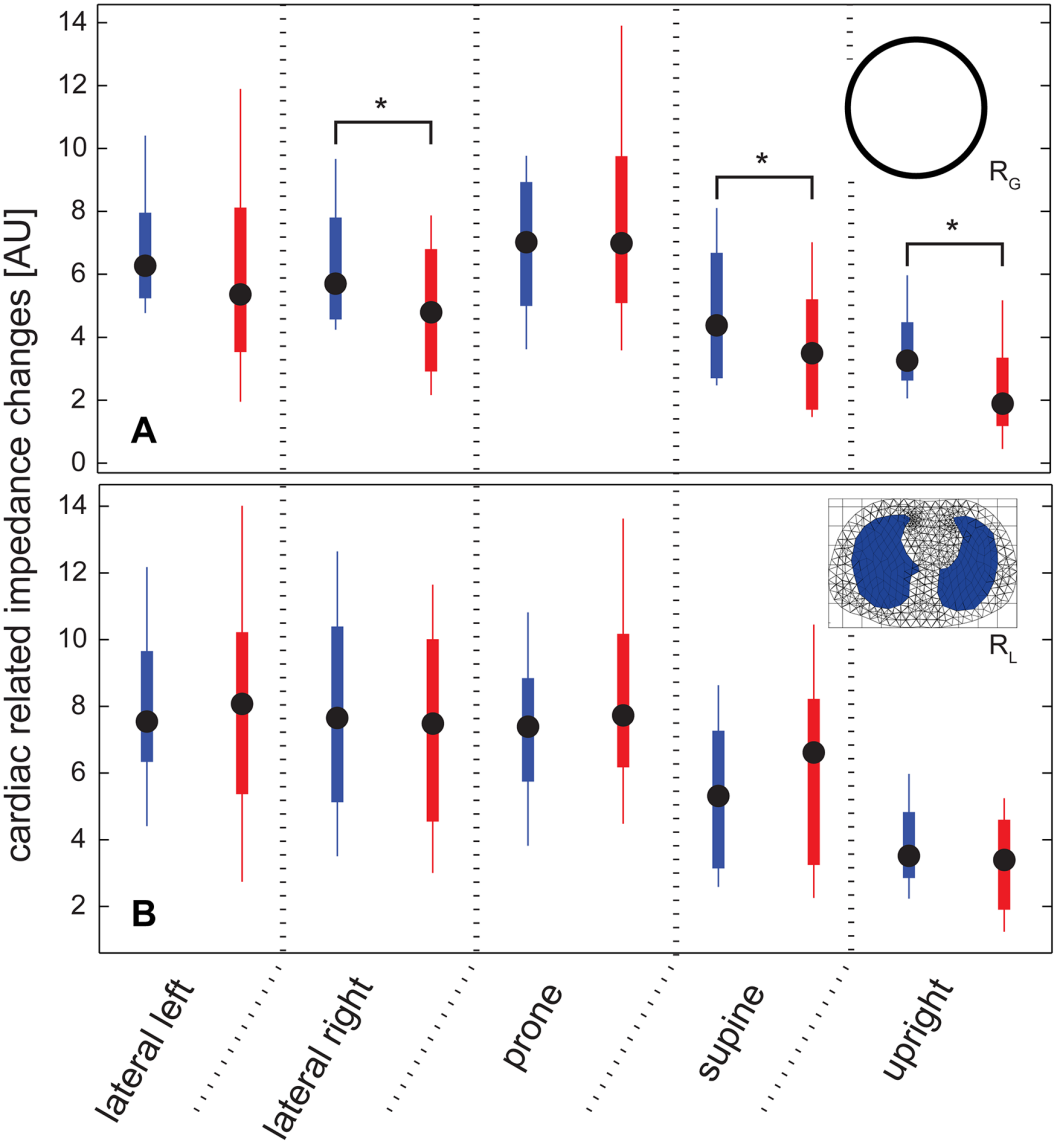
The effect of filtering on the amplitude. A) Comparing ΔZ_CA_ (red) and ΔZ_CR_ (blue) using reconstruction R_G_. Note the significantly lower amplitude obtained with ΔZ_CA_ in upright position. B) Only in prone position a significant difference was found using GREIT reconstructions R_L_ and R_LH_. Here, as an example the values of R_L_ reconstruction are shown. Black circles denote median values, the body box encompasses values between the 25^th^ and 75^th^ percentile. Whiskers cover 95% of the data. * p<0.01.

Using R_G_, R_L_ or R_LH_ we detected significant differences between cardiac related impedance changes ΔZ_CR_ and ΔZ_CA_ for supine position (Wilcoxon signed rank test). Furthermore, using R_G_ a significant difference was found in right-lateral position. Using the GREIT reconstruction containing uniform conductivity (R_0_) resulted in a significant difference between ΔZ_CR_ and ΔZ_CA_ in prone position (Fig 3).

### Respiratory related impedance changes

Tidal volumes measured with the spirometer varied significantly between subjects but yielded no significant differences between body positions (Friedman test), i.e. tidal volumes were constant over different positions.

**Fig 4:**
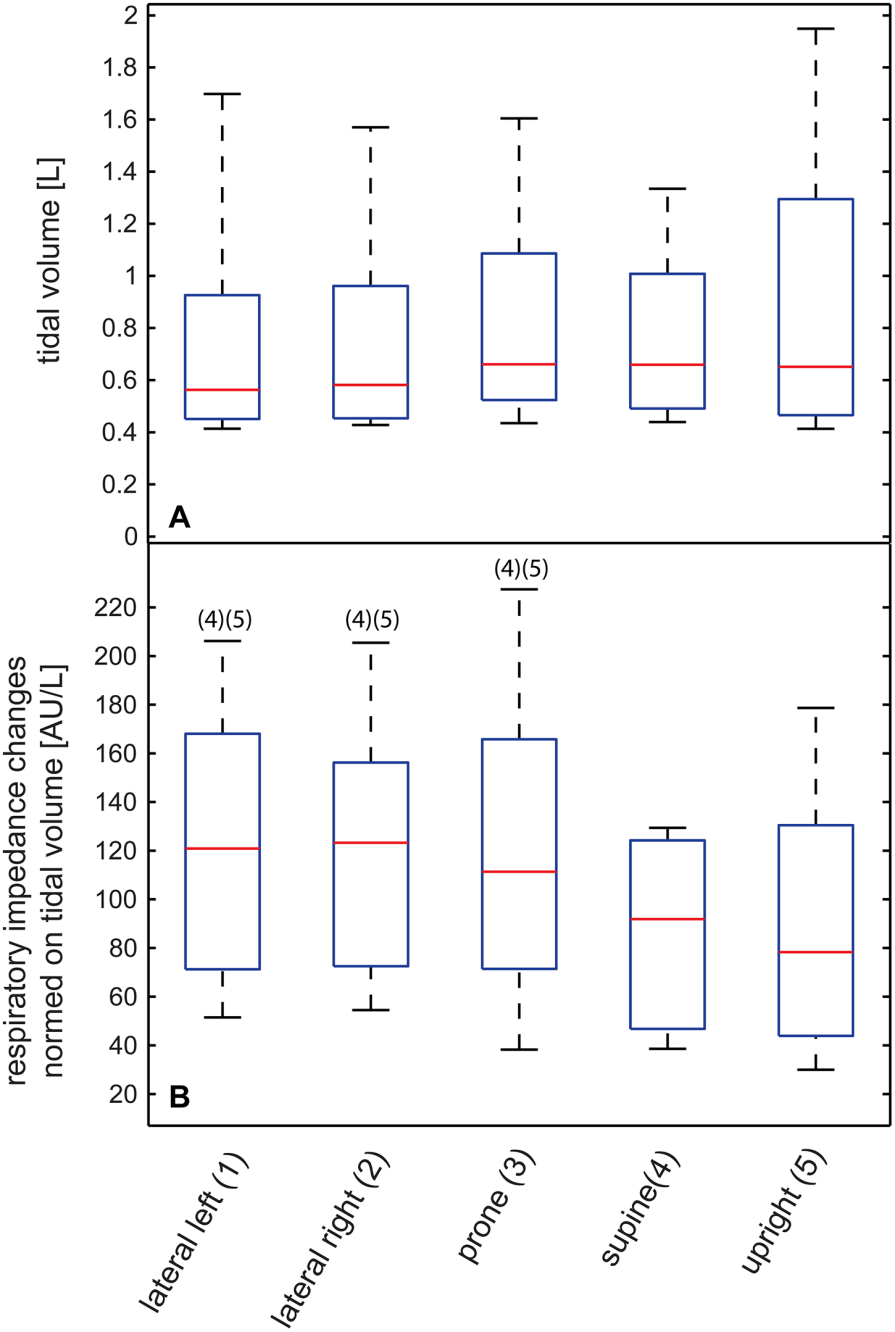
Tidal volumes and respiratory related impedance changes. A) Tidal volumes in liters. B) Amplitude changes due to respiration were normalized to tidal volume [AU/L]. The example shows data reconstructed using R_G_. The red lines denote median values, the blue box encompasses values between the 25^th^ and 75^th^ percentile. Whiskers cover 95% of the data. Positions that are significantly different (Friedman’s test) are labeled with their appropriate number.

Concerning EIT amplitudes upright position was significantly different from left (R_0_, R_L_ and R_LH_), right (R_0_, R_L_ and R_LH_), prone (R_0_, R_L_ and R_LH_) and supine (R_0_). Fig 4 shows the results obtained using R_G_ reconstruction where no differences could be detected.

## Discussion

### Summary

We present evidence that the amplitudes of cardiac related impedance changes are dependent on body position and also on the reconstruction algorithm used. The demonstrated differences are not explained by physiological changes of the cardio-vascular system alone. Especially supine and upright positions presented significantly lower amplitudes. In contrast to a recent publication of Zhao et al. [24] we do find significant differences between the most common reconstruction methods (R_0_, R_G_ and R_L_).

### Cardiac related impedance changes

Cardiac related impedance signals within the lung are often called “pulmonary perfusion” in the literature. It is, however, very controversial whether perfusion is actually the source of this signal. Both, the size of the microvascular bed [25] and stroke volume [26] were linked to the amplitude of cardiac related impedance changes. Considering the observed impedance changes depend on cardiac output, i.e. stroke volume. The more the heart muscle is stretched during filling, the greater is the quantity of blood ejected [27]. Gravity is thus a great determinant of stroke volume, e.g. a recumbent position removes hydrostatic pressure effects and facilitates filling of the heart and thus increases stroke volume. Studies showed that changing from recumbent to upright position can decrease the stroke index up to 40% by affecting venous return [28]. To keep cardiac output constant, changes in stroke volume will therefore lead to changes in heart rate. The gravity dependence of the stroke volume could thus explain parts of the differences between the recumbent and upright positions, but not in supine position. We observe decreases in ΔZ_CR_ in upright position of up to 50% with respect to lateral positions (Fig 2). Given the fact that there was no relevant correlation between the change in heart rate and the change in amplitude from upright to recumbent position and under the assumption that cardiac output did not significantly change in between body positions this cannot be the sole explanation for the differences in EIT amplitude in different body positions.

It is possible, that the weight of the heart compresses the pulmonary microvascular bed and thus decreases cardiac related impedance changes. In prone position, a great part of the weight of the heart is resting on the sternum, leaving the microvascular bed untouched and thus allowing the highest perfusion. Effectively, we can see similar or higher impedance amplitudes for supine positions compared to left or right lateral. Both left and right lateral sides would foreseeably provoke similar effects on microvascular compression. Indeed, we do not see any differences in impedance amplitude when the more recent reconstruction algorithms are used. The supine position leaves the heart resting on some parts of the lungs and thus compresses the microvascular bed most. As a matter of fact, we observe decreases in impedance amplitudes in supine position. Arguably, in the upright position the gravitational force on hydrostatic pressure is greater than the effect due to the weight of the heart.Several other physiological mechanisms occur when changing body position. Changes in intrathoracic pressure might also affect the microvascular system and potentially lead to different EIT amplitudes. Furthermore, the size of the large vessels is possibly variable in different body positions, as well leading to differences in cardiac-related amplitudes measured by EIT. Differences of the shape, position and movement of the diaphragm and consequently the abdominal organs will influence the distribution of cardiac-related impedance changes as suggested by Ericsson and colleagues [8]. In their publication they present similar position-dependent differences in amplitudes compared to our study, unfortunately without discussing these findings.

We propose two main lines of explanation: First, the size of the microvascular bed defines the size of impedance changes. Secondly, stroke volume determines the amount of impedance change. Both try to explain the effect seen in cardiac related impedance changes. It is very likely that both effects exist in reality. It is, however, unclear whether EIT and our analysis set-up pick up these effects.

### Respiratory related impedance changes

Most of the above-mentioned hypotheses cannot explain the differences we observed in respiratory related impedance. Tidal volumes measured with the spirometer were unchanged for different postures. EIT only measures volume changes within parts of the lung which might be different in different body positions. Effects like diaphragmatic and abdominal wall movements will change in different body positions and therefore potentially influence the EIT signal especially with the electrodes placed relatively low like in our study [8]. This effect would be greatest in prone position. We found no differences between all the recumbent positions which cannot completely be explained with this phenomenon. Additionally, differences were not consistent between the reconstruction algorithms. This observation points to a general technical issue regarding EIT signal acquisition or at least image reconstruction.

### Potential back-projection issues

A major issue in EIT image reconstruction is the frame or period the measurement is referenced to. Depending on the parameters that are compared (amplitude, end-expiratory lung impedance,…), a different reference frame might be necessary. We therefore included two reference methods in our study. One would argue that for the comparison of amplitudes a reference frame within the same body position needs to be defined. Except for one reconstruction algorithm we could demonstrate the same main outcome, independent of the reference frame. The more recently developed reconstruction algorithms seem to be less sensitive to the “wrong” reference frame.It is very likely that both cardiac- and respiratory changes are at least partly subject to the same effect. An alternative explanation, taking into account observations from both sources of impedance changes is thus needed.

Grychtol et al. created sensitivity maps of an R_LH_ reconstruction computationally [13]. These sensitivity maps correspond to the amplitude response of the GREIT reconstruction [14]. They revealed lowest sensitivities at the location of the heart and highest on the dorsal side of the left lung. It seems like having a higher a priori conductance on the ventral side of the body would lead to amplified effects on the impedance changes, i.e. the ventral side would be more sensitive to impedance changes. This combined with the fact that ventilation and potentially also perfusion is greater on the dependent part of the lung might explain differences in supine and prone position, which we do see in R_0_, R_L_ and R_LH_. Still, this does not explain the differences seen between recumbent and upright positions. In a further publication, the same group showed that the choice of reconstructed tissue properties will affect the resulting images [29]. We could demonstrate an increase of impedance change in prone position compared to supine when using R_0_, R_L_ or R_LH_. Seeing this difference in all three reconstruction algorithms makes it very unlikely that our findings can be explained only by having chosen the wrong a priori conductance values (Fig 2B–2D).

### Filter effect on amplitude

We were able to quantify the effect of frequency filtering by comparing those values with raw data from apnea. We did not find a consistent pattern of differences due to filtering in the different reconstruction algorithms. In prone position filtering decreases the amplitude for one reconstruction method (R_L_). Given the fact, that the change in amplitude due to filtering is very inconsistent, it is very unlikely that this is the reason for positional differences. Our analysis was spatially restricted to the lung.

### Limitations

All subjects were at rest during the whole acquisition session (< 1 hour). We assumed stable cardio-vascular conditions during data acquisition, but did not measure pulse or stroke volume to confirm this assumption. We see however no reason why cardiac output should significantly change in recumbent positions during such a short period of time. Furthermore, using spirometry we showed that pulmonary activity did not change between postures. There were significant differences in tidal volumes between subjects suggesting that not all subjects were studied during quiet tidal breathing. Review of the individual results showed one subject breathing at significantly higher tidal volumes. There was no change in differences between body positions and reconstruction algorithms with or without this subject. We proposed a novel way to exclude impedance changes related to the activity of the heart. For our analyses we only used the lung region. A possible source of error could be the algorithm used for the spatial separation of heart and lung region. We compared the automatically generated heart masks over the triplicate. The location and the size of the heart were not only consistent through the repetitions, but also through the different postures.

### Relevance

The results of this study might have several consequences for future EIT studies. Especially researchers studying perfusion or V/Q have to be aware of the possibility of positional effects when comparing results taken in different body positions. More research is needed to understand the source of these effects. It is possible that position-dependent reconstruction parameters need to be developed to counteract positional effects.

## Conclusions

The amplitudes of cardiac and respiratory related impedance changes depend on body position and reconstruction algorithm in healthy adults. Prone, supine, upright, left and right lateral positions were investigated. Significant differences in cardiac related impedance amplitudes not fully explained by physiological mechanisms were observed between different postures. The nature of these changes is not yet elucidated, we presented possible explanations that need further testing.
